# LLMs in Patient-Centric Program Evaluation: Addressing Concerns and Further Considerations

**DOI:** 10.1097/PR9.0000000000001352

**Published:** 2025-12-31

**Authors:** Kendra H. Oliver, Leah Russell Flaherty

**Affiliations:** a Responsible Data Science, Office of the Provost, University of Pittsburgh, Pittsburgh, PA, USA; b ArtLab Studio LLC, Pittsburgh, PA, USA; c Allegheny Health Network, Pittsburgh, PA

We sincerely appreciate insightful comments of Hinpetch Daungsupawong and Viroj Wiwanitkit regarding our article.^2^ Their feedback brings important attention to the emotional and cultural dimensions of patient interactions, which we acknowledge as critical considerations when applying large language models (LLMs) in clinical analysis.

LLMs like ChatGPT are trained on vast and diverse datasets, which may not encompass the nuanced emotional and cultural contexts present in patient interactions. Recent studies have explored application methodologies to enhance LLMs in qualitative research, specifically regarding calibration through prompt construction. For instance, integrating expert-guided constraints and automated response validation steps may improve the accuracy of LLMs in extracting and clustering relevant responses from interview transcripts.^5^ In addition, using chain-of-thought reasoning, where the model provides a rationale for its coding decisions, has been shown to significantly improve coding fidelity, aligning more closely with human interpretations.^1^

We must acknowledge that bias and misunderstanding are factors quite common to the human experience, even in clinical settings. Bias and misunderstanding are inherent aspects of both human cognition and artificial intelligence systems and can significantly affect decision-making processes.^3^ Cutting edge technologies to mitigate these biases are in development to make medical LLMs safer and more reliable in health care applications.^4^

With humans and with LLMs, we can work to minimize bias with training and education; yet as responsible data scientists we must be intentional about assessing and validating the output provided by LLMs. Bias plays into clinical decisions shaped by many complex human factors, such as personal experience, patient compliance, ethical beliefs, and cognitive bias. Within the context of our publication, using our knowledge, skills, and understanding of pain psychology and outcomes evaluation, we were able to assess when specific data points did not “fit” into the themes proposed by the LLM. As a team, we offer diverse expertise and yet when presenting these assessments to each other, we were in alignment on lack of fitness as noted in the original publication.

Most importantly, program directors should approach LLM tools with accountability rather than fear. Although we do not present a fully refined methodology, we suggest that, when used thoughtfully, existing technologies can enhance the patient experience. As LLMs continue to be trained to capture greater specificity and nuance, we see an opportunity to leverage these advancements to critically assess and refine current practices. There is no doubt that future LLMs will better incorporate these variables into their analyses. A direct comparison between a qualitative research team conducting thematic analysis and an LLM performing the same task would provide valuable insights for this field. Nevertheless, we propose that current LLM tools, when applied thoughtfully and responsibly, can already serve as useful aids in program evaluation.

## Disclosures

The authors have no conflict of interest to declare.
